# Modelling plant disease spread and containment: Simulation and approximate Bayesian Computation for *Xylella fastidiosa* in Puglia, Italy

**DOI:** 10.1371/journal.pcbi.1013539

**Published:** 2025-10-03

**Authors:** Daniel Chapman, Flavia Occhibove, James M. Bullock, Pieter S. A. Beck, Juan A. Navas-Cortes, Steven M. White

**Affiliations:** 1 Biological and Environmental Sciences, University of Stirling, Stirling, United Kingdom; 2 UK Centre for Ecology & Hydrology, Wallingford, United Kingdom; 3 European Commission, Joint Research Centre (JRC), Ispra, Italy; 4 Department of Crop Protection, Institute for Sustainable Agriculture, Spanish National Research Council (CSIC), Córdoba, Spain; The University of Melbourne, AUSTRALIA

## Abstract

Mathematical and computational models play a crucial role in understanding the epidemiology of economically important plant disease outbreaks, and in evaluating the effectiveness of surveillance and disease management measures. A case in point is *Xylella fastidiosa*, one of the world’s most deadly plant pathogens. Since its European discovery in olives in Puglia, Italy in 2013, there remain key knowledge gaps that undermine landscape-scale containment efforts of the outbreak, most notably concerning the year of introduction, the rate of spread, dispersal mechanisms and control efficacy. To address this, we developed a spatially explicit simulation model for the outbreak spreading among olive groves coupled to a simulation of the real surveillance and containment measures. We used Approximate Bayesian Computation to fit the model to surveillance and remote-sensing infection data, comparing the fits for three alternative dispersal mechanisms (isotropic, wind and road). The model accurately explained the rate and spatiotemporal pattern of the outbreak and found weak support for the wind dispersal model over the isotropic model. It suggests that the bacterium may have been introduced as early as 2003 (95% CI [2000, 2009]), earlier than previous estimates and congruent with anecdotal evidence. The isotropic model estimates the pathogen is spreading at 5.7 km y^-1^ (95% CI [5.4-5.9]) under containment measures, down from 7.2 km y^-1^ (95% CI [6.9-7.5]) without containment measures. Our estimate of an approximately 10-year lag between introduction and detection highlights the need for stronger biosecurity and surveillance for earlier detection of emerging plant pathogens. The outputs from simulations without any disease management also suggest that while containment measures have caused some slowing of *X. fastidiosa* spread, stronger measures will be required to contain the outbreak fully.

## Author summary

Mathematical and computational models are crucial role for understanding plant disease outbreaks and evaluating the effectiveness of disease management. A case in point is *Xylella fastidiosa*, one of the world’s deadliest plant pathogens. Since its European discovery in olives in Puglia, Italy in 2013, there remain key knowledge gaps that undermine landscape-scale containment efforts of the outbreak, most notably concerning the year of introduction, the rate of spread and control efficacy. We developed a simulation model for the outbreak coupled to a simulation of surveillance and containment measures. We used Approximate Bayesian Computation to fit the model to surveillance and remote-sensing infection data. The model suggests that the bacterium may have been introduced earlier than previous estimates and that containment measures may have had some impact on slowing down the spread of the pathogen. Our results highlight the need for stronger biosecurity and surveillance for earlier detection of emerging plant pathogens, and suggest that stronger measures will be required to contain the *Xylella fastidiosa* outbreak fully.

## Introduction

*Xylella fastidiosa* is one of the world’s most deadly plant pathogens [1]. This bacterium has a wide host range of over 690 plant species [2], is vectored by xylem-feeding insects [1,3] and has a significant impact on global agriculture and horticulture [4–7]. *X*. *fastidiosa* is a xylem-limited gram-negative bacterium and the recognised agent of a number of severe and economically important diseases, including Pierce’s disease of grapevines, citrus variegated chlorosis, almond leaf scorch, and other disorders of perennial crops and landscape plants [8]. If the bacterium inoculates susceptible host plants there is a long asymptomatic period [9–11]. Subsequently, symptoms are expressed such as leaf marginal necrosis, leaf abscission, dieback, and plant death through the obstruction of the xylem and a lack of sufficient water flow through the host [1]. To date, there is no cure for infected plants in open field conditions, and the only effective response is to fell diseased trees to prevent further transmission [12,13].

Once restricted to the Americas, a new invasive strain *X. fastidiosa* subsp. *pauca* ST 53 was discovered near Gallipoli, Puglia, Italy, in October 2013 [14] and identified as the causal agent of olive quick decline syndrome [15]. However, symptoms resembling those of *X. fastidiosa* were noticed by olive tree (*Olea europaea*) growers as early as 2008 [16], suggesting a substantial time lag between the bacterium being first introduced and formal identification [3]. A genome-wide analysis revealed that the genotype infecting olive groves in Puglia most likely originated from coffee plants in Costa Rica [17], and that the outbreak was due to a single introduction [18]. Genomic studies also suggested a possible introduction date of 2008 [18,19], but with wide confidence intervals (1952–2015 and 1930–2016, respectively). This introduction date was also suggested by a modelling study [20]. However, given an asymptomatic period lasting approximately 1.2 years [9,10] and the time needed for the pathogen to establish and start spreading, this date appears incongruous with olive growers’ observations of symptoms by 2008 [16] indicating an inconsistency to be resolved.

Since the initial introduction in Puglia, *X. fastidiosa* has spread rapidly. In the infected area a large proportion of the olive trees have been infected [6,16,20] causing millions of tree deaths and resulting in substantial harm to the culture and livelihoods in the region. Control efforts to limit spread have centred around restrictions on moving host plants, surveillance, removal of infected and neighbouring plants and vector control (e.g., Decision [EU] 2015/789, Decision [EU] 2017/2352 and Regulation (EU) 2020/1201) [13,21]. These have been distributed spatially in different demarcated areas comprising the infected zone bordered by containment and buffer zones [13]. Differing intensities of surveillance and control are implemented in each demarcated area, with the aim of containing the spread [22]. Despite these efforts however, spread of the pathogen continues. Modelling studies have shown that the effectiveness of containment strategy is highly sensitive to the width of the containment and buffer zones relative to pest dispersal [12,23–25]. Therefore, model-based assessments of dispersal and spread could improve the efficacy of control measures that involve spatial targeting within an expanding pest range [26,27].

In Puglia, *X. fastidiosa* is mainly transmitted by the polyphagous and widely distributed meadow spittlebug, *Philaenus spumarius* [3,28,29]. Its dispersal capabilities are relatively poorly understood, despite being a key determinant of *X. fastidiosa* spread [29]. Mark-release-recapture (MRR) revealed that 50% of the spittlebug population moved <200 m and 98% < 400 m during the 2-month period of high vector abundance on olives in Puglia [30]. This limited short range vector movement capacity is hard to reconcile with the rapid regional spread of *X. fastidiosa* [20]. However, laboratory flight mill experiments revealed a potential for rarer longer distance *P. spumarius* dispersal, recording a maximum single flight of 5.5 km [31]. In addition, spittlebugs may undergo wind-assisted long-distance dispersal [32] and (anecdotally) human-mediated dispersal by insects “hitchhiking” on vehicles [29,33]. How these dispersal mechanisms determine spatiotemporal patterns of plant disease spread at landscape scale remains an open question that is well suited to computational modelling [34,35].

Previous models of landscape-scale spread of *X. fastidiosa* have also featured very simple representations of dispersal and transmission dynamics [6,23] and also not captured the effect of ongoing surveillance and containment measures [13] on actual and observed disease spread [20]. As such, we argue for more refined model-based estimates of spread using approaches that consider the complex nature of plant disease spread, containment efforts and available surveillance data [36]. This would not only contribute evidence about key mechanisms and parameters for the outbreak but also allow estimation of the efficacy of the control measures designed to contain the outbreak.

Towards this end, we developed a new spatiotemporal stochastic simulation model for the spread of *X. fastidiosa* at landscape scale in Puglia and its containment through surveillance and felling of infected trees [13]. The new model builds on our previous modelling of temporal transmission dynamics within olive groves [10] by incorporating both short- and long-distance dispersal and disease surveillance and felling of infected trees. Using Approximate Bayesian Computation (ABC) [37] we fitted the model to *X. fastidiosa* surveillance data from 2013-2020 and remote sensing estimates of tree loss. Our aims were: (1) to compare models with three different long-distance dispersal scenarios (isotropic dispersal, prevailing wind dispersal and vehicular hitchhiking), hypothesising that accounting for specific vector dispersal mechanisms (wind or vehicles) would improve the fit of the isotropic dispersal model to the data; (2) to estimate the spread rate of the disease and its year of introduction; and (3) to investigate the efficacy of the current containment strategy, hypothesising that the spread rate would be increased in simulations without containment management.

## Materials and methods

**Fig 1:**
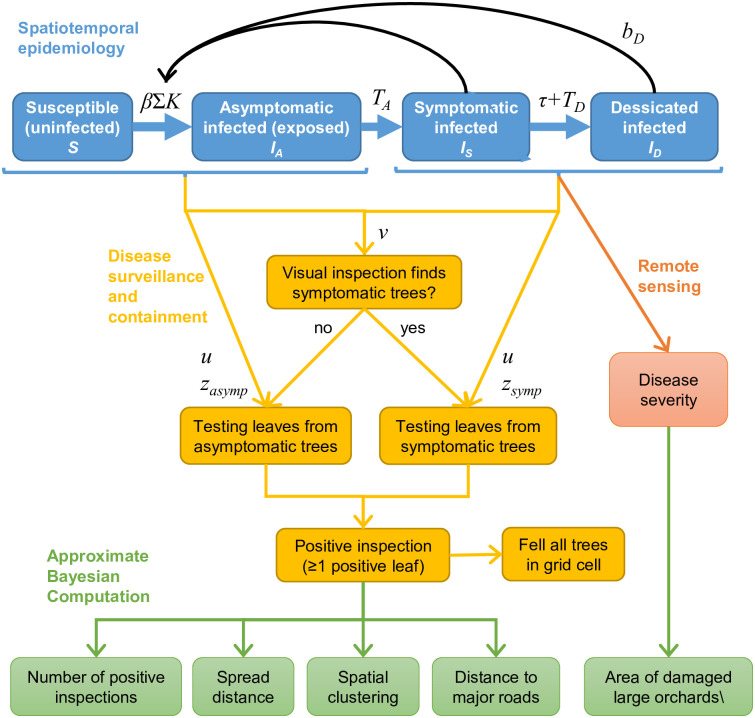
Overview of the model for the *Xylella fastidiosa* outbreak among olive trees in southern Italy. Boxes show the compartments of the epidemiological model (blue boxes), model for the regional disease surveillance and containment (bright orange), remote sensing of severe tree damage (dark orange) and summary statistics produced from the model for fitting to observed data with Approximate Bayesian Computation (ABC) (green). Influences of key parameters and variables are labelled (see main text and Table 1 for explanation).

We developed a spatial epidemiology model in R [38] to simulate spread of *X. fastidiosa* among olive trees in the infected region of Puglia, southern Italy (see Fig 1). The model was based on previously estimated infection dynamics within infected olive groves [10] linked to dispersal models that transmit the pathogen to new locations. In addition, we included a representation of the *X. fastidiosa* monitoring programme and felling of detected infected trees to represent the generation of positive infection reports over space and time, as well as the impact of the containment strategy on disease spread. We used ABC to calibrate model parameters to the monitoring programme as well as estimates of tree loss from remote sensing. This allowed us to evaluate different scenarios for long-distance dispersal and estimate key model parameters.

**Table 1:** Model parameters estimated with ABC, with details of their prior distributions (N = normal with sd being the standard deviation, U = uniform, DU = discrete uniform). The prior for *β*, *T*_*A*_ and *T*_*D*_ was a multivariate normal distribution with covariance (not given here) fitted to posterior estimates from [10].

Parameter	Meaning	Prior distribution	Explanation
*β*	Effective contact rate for transmission	*N*(mean = 19.60, sd = 2.91)	Informative prior based on [10]
*T*_*A*_	Mean asymptomatic period (years)	*N*(mean = 1.176, sd = 0.052)	Informative prior based on [10]
*T*_*D*_	Mean time to desiccation after delay (years)	*N*(mean = 1.331, sd = 0.134)	Informative prior based on [10]
*b*_*D*_	Proportionate infectiveness of desiccated trees relative to symptomatic ones	*U(*min = 0, max = 1)	Full range of possible values
*m*_*short*_	Scaling of short-range distance decay in transmission (km)	*U*(min = 0, max = 1)	Mark-recapture suggests vector lifetime movement capacity <1 km [30]
*m*_*long*_	Scaling of long-range distance decay in transmission (km)	*U*(min = 0, max = 15)	Wide range of possible values, with upper value above estimated *X. fastidiosa* spread rate [20]
*Y*_*0*_	Introduction year	*DU*(min = 1998, max = 2010)	Wide range of possible values, given detection of large numbers of symptomatic trees in 2013.
*v*	Visual inspection inefficiency	*U*(min = 0, max = 0.2)	Wide range of possible values scaling the relationship between number of symptomatic trees and detection probability
*u*	Probability of collecting detectable *X. fastidiosa* in a leaf sample from an infected tree	*U*(min = 0.65, max = 1)	35% of sampled symptomatic trees go on to test negative in the monitoring data, suggesting a lower limit of 0.65.

### Representation of space and time

**Fig 2:**
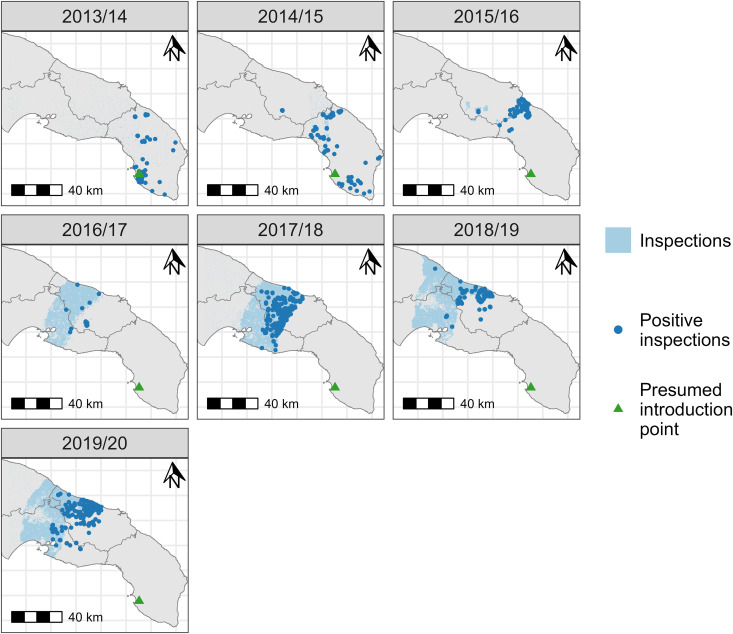
Maps showing the locations of *Xylella fastidiosa* inspections and laboratory-confirmed positive detections carried out in the modelled region and in each model year (May 1^st^ to April 30^th^ of the named years), which were used to determine the locations of inspections in the model simulations. Each inspection covered a 100 x 100 m area, meaning up to four inspections per year in each model grid cell (200 x 200 m). The presumed approximate location of introduction is also shown. The base map is reproduced from the GADM Global administrative areas dataset, under Creative Commons Attribution-ShareAlike 2.0 (https://gadm.org/license.html).

The model simulates *X. fastidiosa* outbreaks with discrete annual timesteps and over a 200 x 200 m discrete grid covering the infected region of Puglia, southern Italy (195,491 grid cells; Fig 2). This grid cell size approximates the median size of olive groves used in our previous modelling of local disease dynamics [10], while reducing the computational demands of a higher resolution grid. The number of olive trees in each grid cell (*N*) was estimated from its proportion cover of olive orchards (*Ω*, see Fig A in S1 Appendix), derived from the InnovaPuglia Spa 2011 land use map (Uso del Suolo – 2011; https://dati.puglia.it/ckan/dataset/uso-del-suolo-2011-uds), and the median olive tree density of 91 ha^-1^ in the olive orchard plots used in our previous study [10]. Based on this, the model assumed a grid cell with 100% olive cover would contain 364 olive trees (i.e., *N* = 364*Ω*).

Model years used May 1^st^ as their start date, corresponding to the approximate start of the adult vector flight period [3]. All simulations initiated the epidemic at the approximate ‘ground zero’ area where tree decline was first detected (Fig 2). However, the year of *X. fastidiosa* introduction (*Y*_0_) was varied as a parameter to estimate with the model.

### Epidemiological model

Within infected grid cells, *X. fastidiosa* transmission and disease progression was simulated with a stochastic and spatial version of an existing discrete-time compartmental model [10]. The structure and parameters of the existing model were estimated to best explain 2–3 year snapshots of disease progression in 17 olive groves [10], justifying the current model compartments and informative priors for several key parameters.

Model compartments are the uninfected susceptible trees (*S*) and three classes of infected trees: asymptomatic (*I*_*A*_, assumed to be non-infective to vectors, so equivalent to the more standard ‘exposed’ or ‘pre-symptomatic’ compartments), symptomatic (*I*_*S*_, assumed to be infectious, so equivalent to the standard ‘infected’ compartment) and desiccated (*I*_*D*_, most foliage is scorched but assumed to still be infectious at a lower level through basal regrowth of green leaves). All trees that become infected progress over time from *I*_*A*_ to *I*_*S*_ to *I*_*D*_ (Fig 1). The transmission model was based on our previous modelling [10], which concluded that asymptomatic trees do not drive transmission but could not rule out the possibility of transmission from desiccated trees, which could occur from any remaining live foliage or basal resprouting (suckers).

During each annual time step of simulations, individual trees progress through these compartments via Bernoulli trials using transition probabilities according to the following equations. The probability of each individual uninfected tree in a grid cell *i* becoming infected in year *t* ($P_{S\to I_{A},i,t}$) depends on the current density of infective trees (i.e., symptomatic or desiccated) in the same grid cell and surrounding areas (Fig 1) formulated as:


$$P_{S\to I_{A,i,t}}=\text{1}-e^{-\beta \sum_{j}\left[K(i,j)\frac{I_{S,j,t}+b_{D}I_{D,j,t}}{N_{j}}\right]}.$$

Parameter *β* is the contact rate for infected olive trees with symptoms and *b*_*D*_ is the relative infectivity of desiccated trees. Since the original model analysis found similar support for a model in which desiccated trees were not infective (equivalent to SEIR) and a model where they were fully infective (equivalent to SEI) [10], here we included parameter *b*_*D*_ as a proportion, reducing the infectiveness of desiccated trees relative to symptomatic trees. To represent infection from multiple locations, *j* indexes the summation over all grid cells in the landscape (including *i*). The function *K* represents pathogen dispersal by scaling a reduction in transmission probability with increasing distance from cell *j* to cell *i* (see below for further details).

Based on our previous modelling [10], the infected asymptomatic trees are assumed to develop symptoms at a constant rate, while desiccation of symptomatic trees is assumed to occur at a constant rate after an initial delay period of *τ* years, representing a minimum time for desiccation. Annual probabilities of individual trees changing from asymptomatic to symptomatic ($P_{I_{A}{\to I}_{S},i,t}$) and symptomatic to desiccated ($P_{I_{S}{\to I}_{D},i,t}$) are:


$$\begin{array}{c} \;P_{I_{A}{\to I}_{S},i,t}=\text{1}-e^{-\frac{\text{1}}{T_{A}}}\\ P_{I_{S}{\to I}_{D},i,t}=\left\{ \begin{array}{c} \text{0}\text{if}y<\tau \\ \text{\textbackslash{}hspace\{-0pt}1\}-e^{-\frac{\text{1}}{T_{D}}}\;\text{if}y\geq \tau . \end{array}\right. \end{array}$$

Parameter *T*_*A*_ is the mean length of the asymptomatic period (years). Variable *y* is the number of years the tree has been symptomatic, *τ* is the desiccation delay parameter (minimum years for desiccation) and *T*_*D*_ is the mean time to desiccation after this delay period. We set *τ* = 3 years in all simulations since in our previous modelling there was very low support for models with other values of *τ* [10].

The dispersal function *K*(*i,j*) represents a decline in *X. fastidiosa* transmission with increasing distance from source *j* to destination *i* (*d*_*i,j*_) which is implicitly linked to vector dispersal [34,39]. We formulated *K* as a mixture of short- and long-distance functions [34], to represent local diffusive-like movements of vectors as well as rarer and less predictable jumps across large distances that can spread pathogens rapidly to new regions [40,41]. Short distance diffusive movement was modelled with a Gaussian function and long-distance dispersal (LDD) with an exponential function:


$$K(i,j)=(\text{1}-L)e^{\frac{{-d}_{i,j}^{\text{2}}}{\text{2}m_{short}^{\text{2}}}}+Le^{\frac{{-d}_{i,j}}{{\text{m}}_{long}}}M(i,j).$$

Parameters *m*_*short*_ and *m*_*long*_ determine the spatial scales of distance decay at both scales. Parameter *L* is the proportion of LDD, which was fixed to a single low number (*L* = 10^-6^). *L* was fixed to avoid confounding the fitting of *L* with that of the scale parameters *m*_*short*_ and *m*_*long*_. We chose this value by “trial and error” to give realistic patchy spread patterns over a wide range of other parameter values.

A further function *M*(*i,j*) was included to represent different LDD mechanisms in a computationally minimal way that did not add extra parameters to estimate. *M*(*i,j*) was formulated to yield a proportion that modifies the amount of LDD from cell *j* to cell *i*, approximating three alternative scenarios:

1. A “basic” scenario with isotropic LDD in which *M*(*i,j*) = 1.
2. A “wind” scenario with anisotropic LDD following prevailing winds in the region, to represent wind-assisted vector flights [32]. We analysed wind directions in the region to derive a function for *M*(*i,j*) giving a value of 1 when the direction from *j* to *i* was aligned to the prevailing wind (approximately southeast), and proportionately lower values when the direction from *j* to *i* was in a less frequent wind direction (Figs B and C in S1 Appendix).
3. A “road” scenario in which dispersing vectors preferentially landed near to major roads as a result of hitchhiking on stationary vehicles in or near olive orchards and then being deposited along the roadside as the vehicle travels [33]. We modelled *M*(*i,j*) as declining from 1 towards 0 with increased distance of the destination grid cell from a major road (as motorways, state highways and provincial roads; see Fig D in S1 Appendix) [33]. We modelled *M*(*i,j*) as declining from 1 towards 0 with increased distance of the destination grid cell from a major road scaled using the short-distance kernel (Fig D in S1 Appendix).

### Containment strategy

Disease surveillance was modelled so that we could compare the spread observed in the database of the official monitoring programme (http://www.emergenzaxylella.it/portal/portale_gestione_agricoltura) with spread patterns produced by the spatial epidemiological model. This was important since spatial variation in the surveillance locations (Fig 2) has a major influence on the observed spread, as opposed to unbiased random surveillance locations. Additionally, tree felling after positive inspections [13] may have slowed the spread of the pathogen, necessitating inclusion of these measures in the spread model.

The surveillance model was a simplified representation of the actual regional containment strategy [12,13]. Real surveillance involves visual inspections by professional surveyors of the Regional Phytosanitary Service [13], followed by sampling of trees and laboratory testing for the disease. If any samples test positive for *X. fastidiosa* this triggers felling of all olive trees within a 100 m radius [12,13].

Inspection locations from 2013 to the end of April 2020 (the latest complete year at the time of the study) were derived from records of testing individual geolocated olive trees in the monitoring programme database (http://www.emergenzaxylella.it/portal/portale_gestione_agricoltura). A small number of outlying isolated records were removed from the data as we suspected they may be ‘incidental’ inspections following reported incidental sightings of disease symptoms rather than part of the systematic survey. To define inspections for the model, individual tree GPS coordinates in the database from the same model year were assigned to the 100 x 100 m grid of the surveillance program. As such up to four inspections could occur per year in each 200 x 200 m model grid cell (Fig 2).

In total, the data contained 495,626 inspections of which 2,329 were positive for *X. fastidiosa* (0.47%). The surveillance intensity and spatial pattern changed year on year (Fig 2) as the outbreak progressed and demarcated areas were updated. Initial surveillance was an area-wide low intensity monitoring to delimit the extent of the outbreak (2013/14). Surveillance then focussed on the most northerly part of the infection front (2014/15) and then targeted northerly clusters of infection with the aim of eradicating them (2015/16). Latterly, surveillance focused on containment and buffer zones spanning and extending beyond the moving infection front (2016/17, onwards) with the aim of containing the spread [13].

During each simulated visual inspection by professional surveyors of the Regional Phytosanitary Service, we assumed the probability of detecting visible symptoms increased with the proportion of trees that were symptomatic. As such, the probability of visual detection in an inspection of cell *i* in year *t* was formulated as


$$V_{i,t}=\text{1}-e^{-\frac{\sigma_{i,t}}{v}}$$

where $\sigma_{i,t}=\frac{\left(I_{S,i,t}+I_{D,i,t}\right)}{N_{i}}\backslash )$ is the proportion of trees with symptoms. *v* is a free parameter scaling the inefficiency in visual detection (i.e., small values of *v* increase the probability of detecting rare symptoms).

Following visual inspection, olive leaves are sampled for laboratory testing. The number of trees sampled per inspection depended on whether symptoms had been visually detected and was drawn randomly from the empirical distribution of numbers of trees tested per inspection in the monitoring database (mean of 3.2 trees when symptoms were seen and 1.9 trees when no symptoms were seen). When symptoms were seen, we assumed only infected symptomatic trees were selected for testing. When symptoms were not found, we assumed only randomly-selected asymptomatic trees were testing so the proportion infected is $\frac{I_{A,i,t}}{\left(I_{S,i,t}+I_{A,i,t}\right)}$. We assumed that some sampled leaves from an infected tree might contain no (or undetectably low) levels of *X. fastidiosa*, so introduced a parameter *u* for the probability of sampling a leaf with detectable *X. fastidiosa* load from an infected tree. Finally, we assumed laboratory testing would have a probability of false negative results *Z*, which differs for symptomatic or asymptomatic trees, as testing procedures depend on symptom status and symptomatic trees will likely have higher bacterial load and therefore lower false negatives.

Therefore, the probability of each sample from cell *i* returning a positive result in year *t* ($p_{i,t}^{+}$) was formulated as:


$$p_{i,t}^{+}=\left\{ \begin{array}{cc} u\left(\text{1}-Z_{symp}\right) & \text{if}symptoms\text{visually detected}\\ \frac{I_{A,i,t}}{I_{S,i,t}+I_{A,i,t}}u\left(\text{1}-Z_{asymp}\right) & \text{if no symptoms visually detected} \end{array}.\right.$$

The false negative rates (*Z*) were estimated using Latent Class Analysis on the monitoring data as *Z*_*symp*_ = 0.0512 and *Z*_*asymp*_ = 0.0614 for symptomatic and asymptomatic trees, respectively (Section S1.3 in S1 Appendix). False positives were not included in the model as this analysis found negligible rates of false positives.

Using these probabilities, Bernoulli trials were used to simulate whether each sampled tree was confirmed as *X. fastidiosa* positive. If any tree returned a laboratory-confirmed positive that inspection was deemed positive. Following a positive inspection, all trees in the 200 x 200 m grid cell were felled, approximating the 100 m felling radius used in the actual containment strategy [12,13].

### Approximate Bayesian computation

Approximate Bayesian Computation (ABC) using rejection sampling [37,42] was used for model selection among the three long-distance dispersal scenarios and then for parameter estimation of the chosen dispersal scenario. ABC is based on matching summary statistics from data to those produced by the model rather than using likelihoods [43,44]. It was used since we considered it intractable to produce a likelihood function for the observed spread data derived from the monitoring scheme. Furthermore, ABC is well suited for parameter estimation for complex stochastic models as large numbers of model runs can be run in parallel on high-performance computing clusters [42].

To produce reliable results from ABC, a range of different summary statistics relevant to the pattern of interest should be used [43]. From the monitoring database, we chose the following summary statistics to capture the pattern of spread for each year between 2013 and 2020: number of positive inspections; spread distance (99^th^ percentile distance from introduction point for positive inspections); clustering of positive inspections (local Moran’s *I* on positive grid cells, weighted using a Gaussian moving window with 200 m standard deviation); and association of positive inspections with major roads (median distance of positive inspections to a major road, which could indicate an effect of roads on the spread [33]).

In addition, we obtained independent estimates of the size of the new area with severe damage in each year from 2010 to 2017 from a previous remote sensing study [45]. In that study, remote sensing was able to detect large olive orchards (>12.5 ha) with high levels of desiccation. *X. fastidiosa* progression in olives trees is monitored on a visual severity scale from 0 (unaffected) to 5 (canopy entirely desiccated) [46,47]. In the remote sensing it was estimated that a mean severity score above ~2.9 was detectable [45]. To estimate an equivalent area of severe damage from the model, we first identified the model grid cells corresponding to large olive orchards, and then estimated their mean disease severity score. The modelled severity score of individual trees increased in yearly increments for symptomatic trees, reaching a maximum of 5 for desiccated or felled trees. This aligns with the definition of the severity scores and recorded within cell disease progression [10]. The new area of large orchards with mean severity >2.9 was then calculated for each year of the simulation.

ABC rejection sampling was performed using these summary statistics [43,44]. First, 10^5^ simulations were performed for each long-distance dispersal scenario (basic, wind and road) with parameter values drawn randomly from the prior distributions in Table 1.

For each simulation, a standardised distance *ρ* between the observed summary statistics (*m*_*obs*_) and the summary statistics calculated from simulated outbreaks (*m*_*sim*_) was calculated as


$$\rho =\sqrt{\sum_{k}{\left(\frac{m_{sim,k}-m_{obs,k}}{\sigma_{sim,k}}\right)}^{\text{2}}}$$

where *k* indexes over all the summary statistics and *σ*_*sim,k*_ is the standard deviation of summary statistic *k* among all the simulations. Those simulations with *ρ* less than a certain acceptance threshold (detailed below) are accepted as the best matches to the observed data and their parameter values form the posterior distribution estimates [43,44].

The relative support for the more complex dispersal scenarios (wind and road) was evaluated by calculating Bayes Factors compared to the simpler basic scenario (Section S1.4 in S1 Appendix). Having selected the single dispersal scenario with the greatest support from Bayes Factors, posterior parameters were estimated for the chosen dispersal model. To improve the estimation, a further 4x10^5^ simulations were performed for this model, giving a total of 5x10^5^ simulations on which rejection sampling was implemented. An acceptance threshold of 0.0002 was used giving a posterior sample of 100 simulations [42].

The accuracy of the parameter estimation was assessed using a leave-one-out cross validation (LOO CV) procedure [42,43]. For this, a randomly selected simulation was used as the data for parameter estimation (i.e., its simulated summary statistics were used as *m*_*obs*_), rejection sampling was performed with the remaining simulations and the estimated parameter values were compared to the true values used for the focal simulation. We ran LOO CV 10,000 times with randomly chosen focal simulations, to calculate both the RMSE for the parameter estimates and the proportion of parameter estimates whose 95% credible interval contained the true values.

### Containment modelling

Finally, the calibrated model was used to simulate spread under the current containment strategy, where trees are felled after positive detections, and for a “no containment” scenario with no felling performed. One simulation was run for each of the 100 posterior samples from the ABC, and the results were averaged to estimate pathogen spread for both scenarios in terms of the total number of infected trees, number of felled trees and spread distance (quantified as the 99^th^ percentile distance of infected trees from the origin point).

## Results

### Comparison of dispersal scenarios

The Bayes Factor analysis (Section S.2.1 in S1 Appendix) found lower support for the highly simplified ‘road’ long-distance dispersal model than the ‘basic’ model with isotropic long-distance dispersal. The ‘wind’ long-distance dispersal model was slightly better supported than the ‘basic’ model, but not sufficiently so to select it over the conceptually-simpler basic model. Therefore, the basic model was used in the remainder of this study.

### Parameter estimation for the basic dispersal model

**Fig 3:**
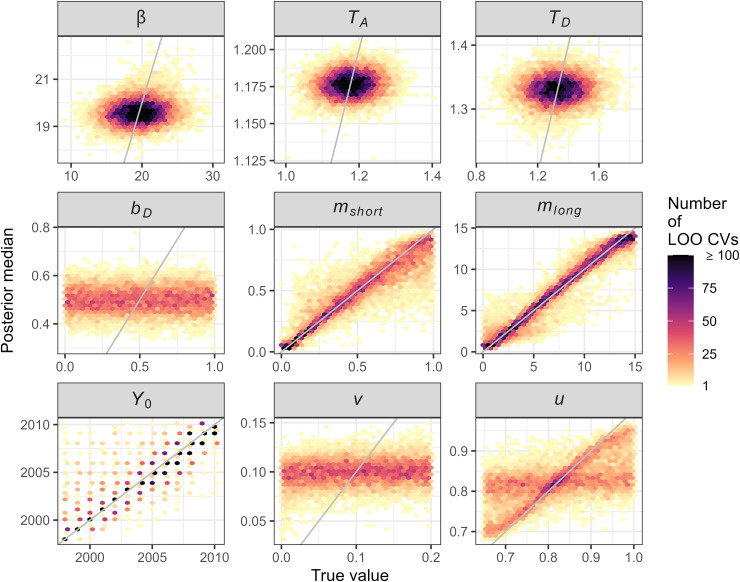
Accuracy of parameter estimation estimated with 10,000 leave-one-out cross validations (LOO CV) of the Approximate Bayesian Computation rejection sampling and acceptance threshold of 2x10^-4^. Background shading shows the number of cross-validations. Where the highest densities of values align to the grey 1:1 lines, this indicates that parameter estimation is reliable. See Table 1 for full parameter explanations: *β* = transmission rate; *T*_*A*_* =*_* *_asymptomatic period (years); *T*_*D*_ = desiccation period (years); *b*_*D*_ = infectiveness of desiccated trees; *m*_*short*_ = short-range dispersal distance (km); *m*_*long*_* = *long-distance dispersal distance (km); *Y*_*0*_ = Introduction year; *v* = visual inspection inefficiency; *u* = leaf sampling probability.

Leave-one-out cross validation (LOO CV) of the rejection sampling on the basic model showed that ABC parameter estimation was reliable for the two dispersal parameters and introduction year, shown by a 1:1 relationship between the parameter value and its estimate (Fig 3, panels for *m*_*short*_, *m*_*long*_ and *Y*_*0*_). The LOO CV showed ABC was not effective at estimating the three disease dynamics parameters with highly informative priors (*β*, *T*_*A*_, and *T*_*D*_), but this was not a concern as we already had good estimates of these from our previous study [10] and used informative priors. The infectivity of desiccated trees (*b*_*D*_) and two inspection parameters (*v* and *u*) were also estimated poorly, although for *u* the LOO CV yielded accurate estimates in about half of the cases (Fig 3). These parameters had uninformative priors capturing a wide range of plausible values, and so even though we could not estimate them, the posterior simulations captured a wide range of uncertainty in their values.

**Fig 4:**
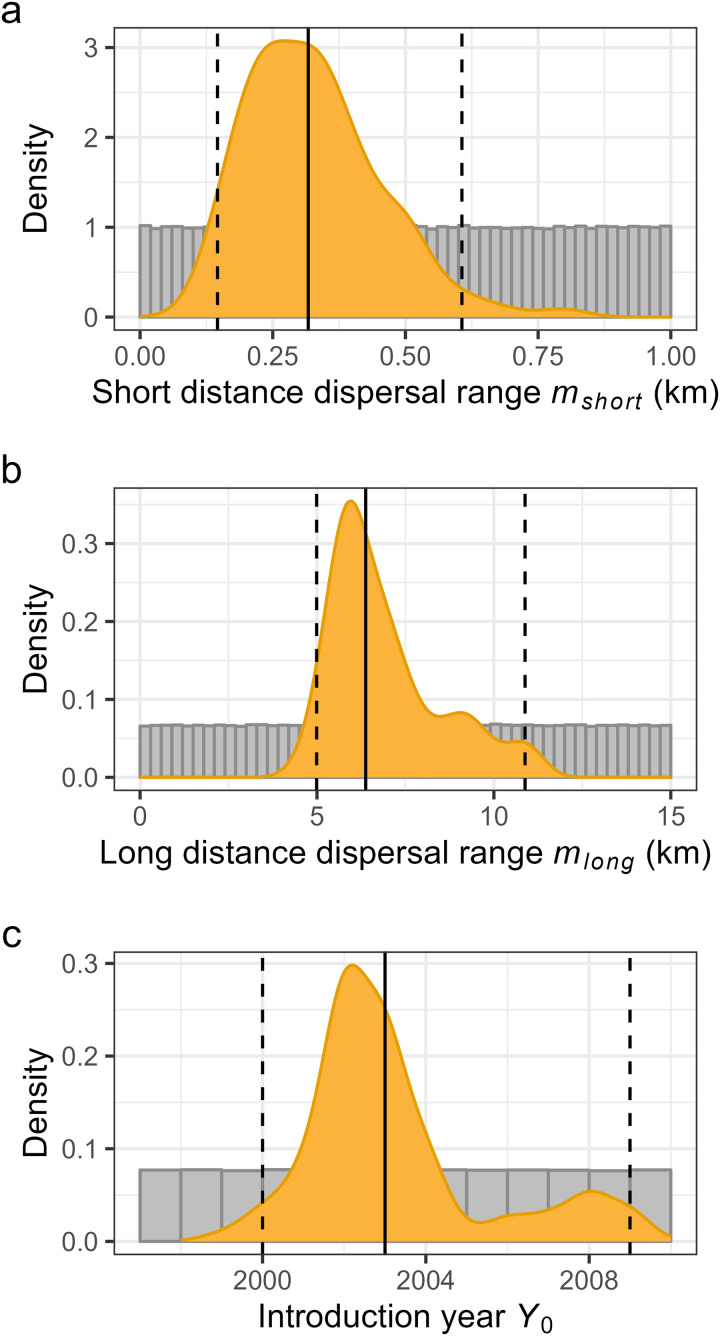
Posterior estimates for the three identifiable parameters in the basic model shown as kernel density plots with vertical lines showing the medians (solid lines) and 95% credible intervals (dashed lines). Grey background histograms show the uniform prior distributions.

Posterior distributions of the three identifiable parameters are shown in Fig 4. The median estimates for the short- and long-distance dispersal ranges were 0.317 km (95% CI [0.146, 0.606]) and 6.376 km (95% CI [4.990, 10.881]), respectively. The median estimate of the introduction year *Y*_*0*_ was 2003 (95% CI [2000, 2009]), although the posterior was somewhat bimodal.

### Posterior simulations

**Fig 5:**
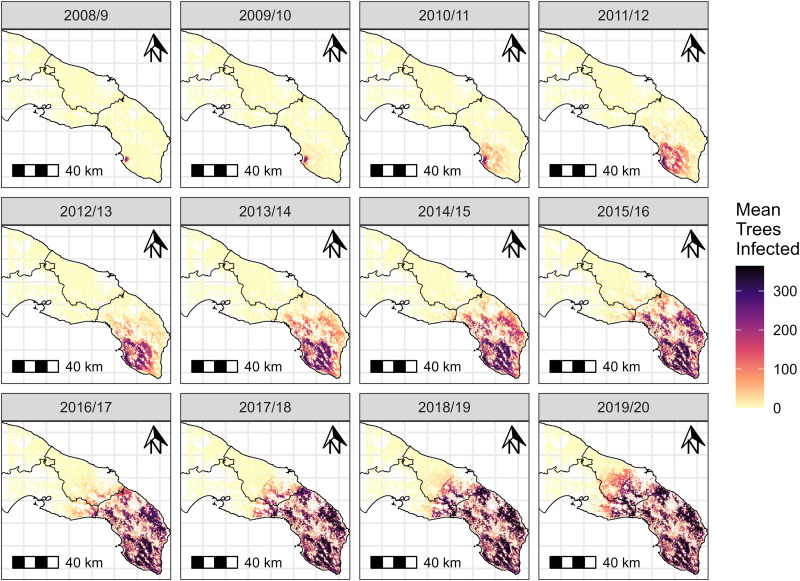
Average model *Xylella fastidiosa* spread, shown as the mean number of infected trees (*I*_*A*_^* *^+ *I*_*S*_ + *I*_*D*_) per grid cell at the end of each modelled year in single simulations with each of the 100 posterior parameter values. Simulations started at the estimated introduction year for each posterior parameter, but only 2008/9 onwards is shown as there was little earlier spread. For scale, the grey background grid represents 20x20 km divisions. The base map is reproduced from the GADM Global administrative areas dataset, under Creative Commons Attribution-ShareAlike 2.0 (https://gadm.org/license.html).

Using these parameter estimates, posterior predictive checks indicated a good correspondence between observed and simulated summary statistics (Fig F in S1 Appendix). The main exception was that the model over-predicted the number of positive inspections in 2017/18. The average modelled spread pattern produced from these parameters is shown in Fig 5, noting that individual simulations produce a patchier and more stochastic spread pattern than the smoother average of many simulations shown in Fig 5 (see Fig G in S1 Appendix for an example). This was especially so at the invasion front, where new infection foci are seeded and grow.

### Management scenario simulations

**Fig 6:**
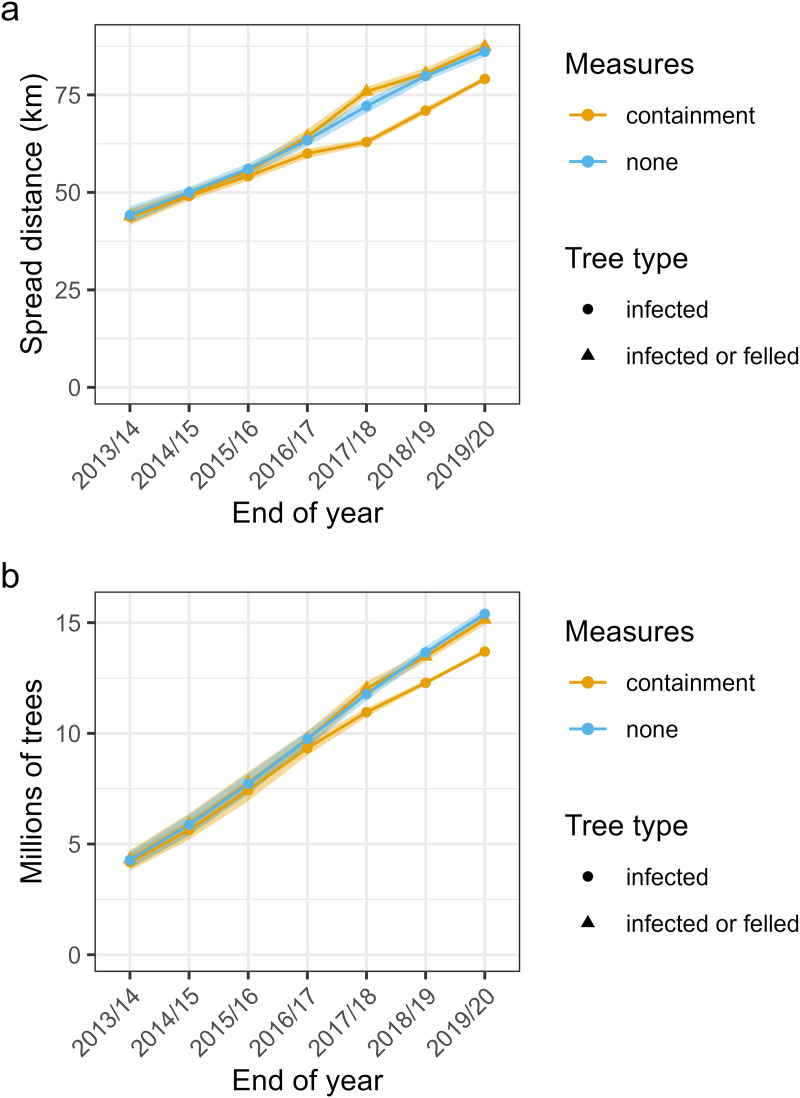
Modelled effect of containment measures on spread of *Xylella fastidiosa* in 100 simulations from the posterior model parameters. Panels show the mean (a) trees infected and felled and (b) 99^th^ percentile spread distance. Also shown is a “none” scenario in which no felling is applied after positive infection detections. Ribbons are bootstrapped 95% confidence intervals for the means.

Simulations in which no felling occurred were compared to the default model of felling after positive inspections (Fig 6). Linear regression of the spread distance for infected trees against year gives an estimated spread rate of 5.7 km y^-1^ (95% CI [5.4, 5.9]) between 2013 and 2020 when felling was applied. This compares to 7.2 km y^-1^ (95% CI [6.9, 7.5]) without felling. This reduction in modelled *X. fastidiosa* spread rate appears to have been driven by a large amount of felling in the model in the year 2017/18, when there were high numbers of positive inspections and felling (Figs 6 and Fig F in S1 Appendix). Further, jointly considering the spread rate of both the infected and felled trees (7.6 km y^-1^, 95% CI [7.3, 7.9]) there was similar spread to the simulation with no felling. Therefore, modelled felling overall had only a small influence on simulated pathogen spread, in terms of distance from the origin, and reductions in trees lost to infection were largely countered by losses from felling (Fig 6).

## Discussion

We developed a spatial epidemiological model for the spread, surveillance and containment of *Xylella fastidiosa* in olives in Puglia, southern Italy, and used a novel approach to estimate model parameters from the surveillance data using Approximate Bayesian Computation (ABC) [44]. This allowed us to compare the support for alternative long-distance dispersal mechanisms for the vector, finding that wind and road dispersal were not clearly better explanations of the observed spread than basic isotropic dispersal. It also allowed us to estimate important epidemiological parameters such as the ranges of short- and long-distance dispersal and the year of introduction.

Modelling *X. fastidiosa* spread at landscape scale is difficult due to missing knowledge about key dynamic processes such as vector dispersal and transmission [36,48] and the spatially biased surveillance data from which spread is observed. Different sources of data are available and can provide different information about the epidemic [49]. By modelling surveillance alongside pathogen spread and fitting the model to summary statistics from both the surveillance data and remote sensing of tree loss, we were able to evaluate mechanisms and parameters determining the rate of spread and the efficacy of measures applied to contain the outbreak.

Estimating the long-distance dispersal of *X. fastidiosa* is critical since this directly affects the efficacy of containment and eradication strategies [12,23]. If the buffer zones are too narrow compared to pathogen dispersal, then new infection foci will appear beyond the buffer zone, impeding management of the outbreak. Additionally, better understanding of dispersal mechanisms could be used to target surveillance and biosecurity measures. Therefore, we compared models based on simplified representations of three different modes of long-distance dispersal considered important for the outbreak. We found some support for anisotropic long-distance dispersal in the prevailing wind direction compared to the basic isotropic model, which is consistent with suggestions of wind-assisted long-distance flights of the *X. fastidiosa* vector *P. spumarius* [31,32] and studies of other vectors of plant diseases [32,50].

By contrast, our model of long-distance pathogen dispersal following the major road network was much less supported, despite vehicle hitchhiking being proposed as an important mode of *X. fastidiosa* spread [29,33]. In part, this may reflect the simplistic representation of hitchhiking in the model, including the restriction of dispersal to the major road network with no representation of the denser network of minor roads. These and other simplifications may have affected our ability to differentiate rare and idiosyncratic long-distance dispersal mechanisms [51,52]. Furthermore, long-distance vector dispersal may be driven by a combination of different mechanisms so future modelling might explore multiple dispersal mechanisms, ideally informed by additional data collection designed to quantify those mechanisms [53]. For example, targeted surveys of spread from isolated infection foci beyond the main infection front could identify downwind or along-road spread [34].

Dispersal estimates from the basic model (isotropic dispersal) were broadly consistent with empirical information on *P. spumarius* movement. Bodino et al [30] found a median movement of 26 m per day in olive groves. Their extrapolation of daily movement using a simple random walk model gave a median net displacement of approximately 200 m over the 2-month main vector transmission period [30]. This is very close to the median displacement that results from our posterior estimate of short range dispersal (for a half-normal distribution with standard deviation of *m*_*short*_ = 0.32 km the median net displacement is 0.674*m*_*short*_ = 0.22 km). Our posterior estimate of the mean long-distance dispersal distance (*m*_*long*_ = 6.38 km) is also consistent with the maximum recorded single flight distance of 5.5 km [31]. Therefore, we suggest that the model provides reasonable and useful estimates of infection spread rates. These could be used to update modelling of future economic impacts of *X. fastidiosa* [6] and refining the size of containment zones and felling radii employed as part of the containment strategy [12].

Another important parameter is the introduction year, estimated to be 2003 (95% CI [2000, 2009]). Previous studies have centred estimates on 2008 [18–20], coinciding with anecdotal reports of growers first noticing symptoms [16]. Our estimate suggests the possibility of an earlier presence of *X. fastidiosa* 5 years prior to symptom observation and 10 years prior to confirmation, which in the model is needed to explain the large size and spatial pattern of the epidemic when first recorded by monitoring. Substantial delays between introduction and detection impede control strategies of this and other invasive species [54]. If the bacterium had been detected sooner, then employing an eradication strategy may have been more feasible [12,55]. This highlights the need for effective and improved international plant biosecurity alongside rapid reporting and detection methods [49].

Simulations compared current containment measures [12,13] to a scenario with no felling. In the model, felling caused a reduction in the overall pathogen spread rate over the monitoring period from 7.2 km y^-1^ to 5.7 km y^-1^. However, this was mostly accounted for by a single year in which the model produced unrealistically high levels of positive inspections and felling. In that year it is likely that the simulated invasion front happened to align strongly to the high surveillance buffer zone used to try to contain the pathogen (Fig 2) [13]. These results should be caveated by their basis in modelling, rather than empirical measurement. However, they could explain why containment of *X. fastidiosa* has proved so difficult [12,16,20].

This suggests that the current containment strategy may be very sensitive to decisions about the position and width of the buffer zone (5–10 km) relative to the infection front and the long-range dispersal of the bacterium [23]. It also suggests that very high rates of surveillance and felling at the invasion front are needed to limit spread. Ideally containment would employ a large and intensively-surveyed buffer zone that encompasses the whole area where long-distance dispersal produces new disease foci, though the cost implications of this could be prohibitive. To investigate this further, the model could be used to explore alternative control policies [23,56,57], such as optimising the location and size of buffer zones, surveillance intensity and sampling procedures. Additionally economic costs of surveillance and production losses from the disease and felling could also be built in to the model, to investigate the cost-effectiveness of alternative strategies [6,57]. These would require additional modelling components beyond the current framework but are important future directions with potential to improve disease management.

Approximate Bayesian Computation has rarely been used to fit spatiotemporal models of plant diseases [58], though it is widely used in other fields [37,43]. An advantage of ABC is that it is likelihood free and well suited to stochastic simulation models [42]. It also allowed us to use data from both the official surveillance program for *X. fastidiosa* alongside remote sensing estimates of damage to olive orchards. A strength of this data fusion is that models draw evidence from datasets that are informative about different aspects of the process being modelled [59]. In our case, the surveillance data was spatially biased but gave valuable information about the spatial pattern of *X. fastidiosa* occurrence, while the remote sensing data provided information on tree decline occurring later in the disease progression.

The model developed here predicts *X. fastidiosa* spread dynamics at regional scale. However, vector dynamics were implicitly represented in the current model, and so a more detailed representation of their densities, dynamics, behaviour and seasonality might give improved estimates of transmission and pathogen spread [60,61]. For example, spatial and temporal variation in vector densities and resulting transmission rates could be estimated with predictive models based on vegetation and weather conditions since *P. spumarius* is more abundant in cooler and moister habitats [62] and its year-to-year population dynamics show lagged effects of weather [63]. Our transmission model could also be modified to account for vector feeding preferences [64] including any avoidance of heavily infected plants [48,60,61]. Additionally, the model does not explicitly represent the persistent infection of *P. spumarius* foreguts by *X. fastidiosa* throughout the adult life stage [3]. This may be an important limitation, since this persistent infection combined with a capacity for repeated bouts of *P. spumarius* movement and feeding among olive trees [30,31] may mean that a single dispersing vector could initiate a relatively large cluster of infected trees in a single year. However, we suggest that such model extensions should be developed based on specific empirical studies quantifying these effects rather than hypothesis or speculation, and tested to determine whether they increase model utility for informing control efforts.

## Conclusions

Our novel approach to calibrating an epidemiological model for *X. fastidiosa* spread in southern Italy accurately predicts the rate and pattern of the outbreak and could provide a valuable tool for prioritising future surveillance to improve containment of the epidemic. The modelling provides useful estimates of dispersal mechanisms, distances and pathogen spread rates, and suggests that the bacterium may have been introduced to Italy as early as 2003, pushing back previous estimates and better aligning with anecdotal evidence. Further, the model suggests that containment measures may have had a limited effect on reducing the rate of spread to date, but could be made more effective by targeting surveillance to (and beyond) the edge of the infection wavefront and rigorously implemented felling after positive infection detections.

## Supporting information

S1 Appendix Modelling plant disease spread and containment: Simulation and Approximate Bayesian Computation for *Xylella fastidiosa* in Puglia, Italy. (PDF)
