# Supplementary material for: Modelling plant disease spread and containment: Simulation and approximate Bayesian Computation for Xylella fastidiosa in Puglia, Italy
Source: PLoS Comput Biol. 2025 Oct 3;21(10):e1013539. doi: 10.1371/journal.pcbi.1013539 (PMC12510647; doi:10.1371/journal.pcbi.1013539)
Supplement: S1 Appendix — (PDF) [file pcbi.1013539.s001.pdf]

S1 Appendix to: Modelling plant disease spread and containment: Simulation and Approximate Bayesian Computation for *Xylella fastidiosa* in Puglia, Italy

Daniel S. Chapman[1,*], Flavia Occhibove[2], James M. Bullock[2], Pieter S. A. Beck[3], Juan A. Navas-Cortes[4] & Steven M. White[2,*]

[1]Biological and Environmental Sciences, University of Stirling, Stirling, UK.

[2] UK Centre for Ecology & Hydrology, Wallingford, UK.

[3]European Commission, Joint Research Centre (JRC), Ispra, Italy.

[4]Department of Crop Protection, Institute for Sustainable Agriculture, Spanish National Research Council (CSIC), Córdoba, Spain

Contents

S1. Methods 2

S1.1. Olive grove cover in the modelled region 2

S1.2. Details of the anisotropic long-distance dispersal kernels 3

S1.2.1. Wind 3

S1.2.2. Road 5

S1.3. Laboratory testing model 7

S1.4. Bayes Factor model selection 10

S2. Results 11

S2.1. Comparison of dispersal scenarios using Bayes Factors 11

S2.2. Posterior predictive checks 13

S2.3. Example spread simulation 14

Supporting References 15

S1. Methods

S1.1. Olive grove cover in the modelled region

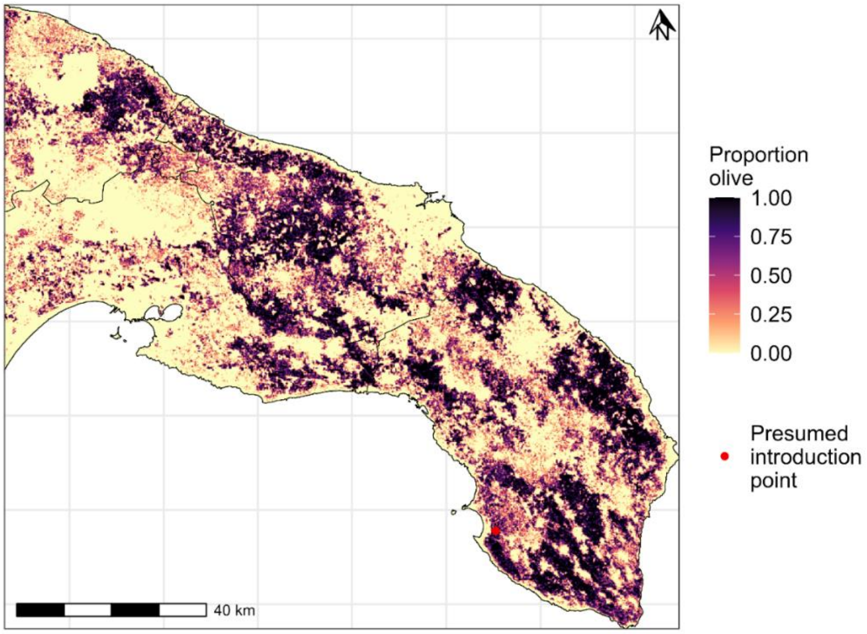

Figure A. *Map showing olive grove cover in the modelled region, used in the modelling to represent the distribution of susceptible host plants for* Xylella fastidiosa*. Olive cover was derived from the 2011 Puglia land use map (Uso del Suolo – 2011) class 223 Ulivetti (https://dati.puglia.it/ckan/dataset/uso-del-suolo-2011-uds), under Italian Open Data License 2.0 (IODL 2.0). The base map is reproduced from the GADM Global administrative areas dataset, under Creative Commons Attribution-ShareAlike 2.0 (https://gadm.org/license.html).*

S1.2. Details of the anisotropic long-distance dispersal kernels

S1.2.1. Wind

In the “wind” scenario, Long Distance Dispersal (LDD) followed the anisotropic distribution of prevailing winds in the region, which may determine insect long-distance flight directions.

Prevailing wind dispersal directions were estimated using the HYSPLIT Trajectory model [1] run from the SplitR R package [2]. HYSPLIT was used to simulate 10 trajectories per day of a wind-borne particle for every day in the main vector flight period (May to October) of 2013 to 2017 (45400 trajectories in total). Trajectories were initiated from random locations in the region and for a particle released at 20 m height, otherwise using all default HYSPLIT settings. Compass directions (clockwise from north) of the simulated trajectories were calculated, resulting in a bimodal distribution of prevailing winds with a peak at around 2.5 radians (145° clockwise from north) and a second peak or shoulder at around 0.5 radians (Fig B).

To represent this as a function, a mixed Von Mises distribution (*V*) was fitted by maximum likelihood to the distribution of directions (θ radians) giving the following equation

$$V(\theta) = pe^{(-\kappa_1 \cos(\theta-\mu_1))}/\left(2\pi I_v(\kappa_1)\right) + (1-p)e^{(-\kappa_2 \cos(\theta-\mu_2))}/\left(2\pi I_v(\kappa_2)\right),$$

with fitted parameters p = 0.6066, κ_1 = 2.2418, μ_1 = 2.5640, κ_2 = 1.0187, μ_2 = 0.3993.

For scaling long-distance dispersal in the model, this function was normalised by its maximum value so that is gave a maximum value of 1 at the modal wind direction, i.e., $M(i,j) = V(\theta_{i,j})/0.3633$. This resulted in LDD being biased in the approximately southeast direction of the prevailing wind (Fig C).

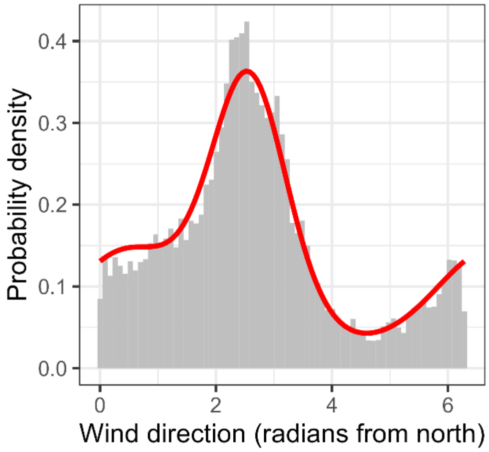

Figure B. *Histogram showing the distribution of HYSPLIT-simulated wind dispersal directions in Puglia during the main vector flight period. The red line shows a mixed Von Mises distribution fitted to this distribution to scale the anisotropic directionality of long-distance dispersal in the model.*

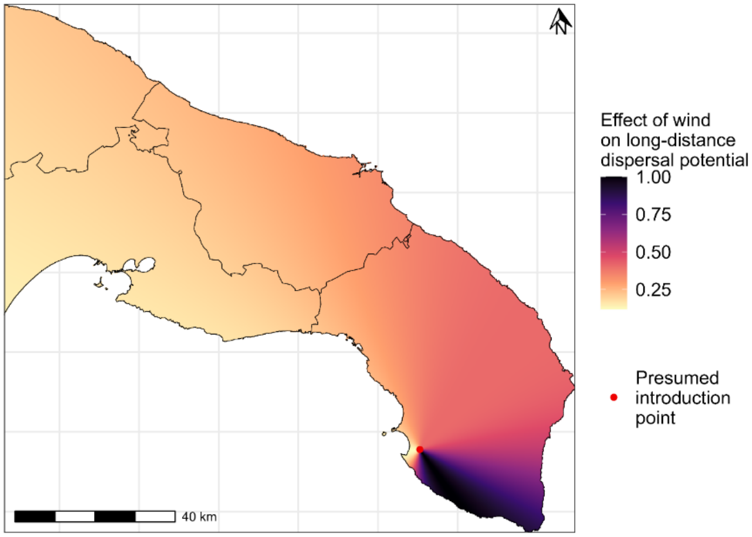

Figure C. *Illustrative effect of the prevailing wind on potential long-distance dispersal from a single example source location, here set at the presumed introduction point. Long-distance dispersal is biased towards a south-easterly direction from sources of infection. The base map is reproduced from the GADM Global administrative areas dataset, under Creative Commons Attribution-ShareAlike 2.0 (https://gadm.org/license.html).*

S1.2.2. Road

In the "road" scenario we assumed dispersing vectors preferentially landed near to major roads [3], defined as motorways (Autostrade), state highways (Strade statali) and provincial highways (Strade provinciali), mapped from Open Street Map obtained through https://www.geofabrik.de/. This is based on a potential LDD mechanism by which vectors settle on stationary vehicles in or near olive orchards and then 'hitchhike' on them as they depart and travel along roads, becoming deposited along the roadside as the vehicle travels at speed [3].

For simplicity, we assumed that deposited vectors would have similar movement capacity to their local movements. Therefore *M*(*i,j*) was formulated as declining with increased distance of the destination grid cell from a major road ($d_{road,i}$) similarly to short-range dispersal Gaussian function in *K*, i.e., $M(i,j) = e^{-d^2_{road,i}/2m^2_{short}}$. While highly simplistic, this had the effect of channelling long-distance dispersal along the major road network (Fig D).

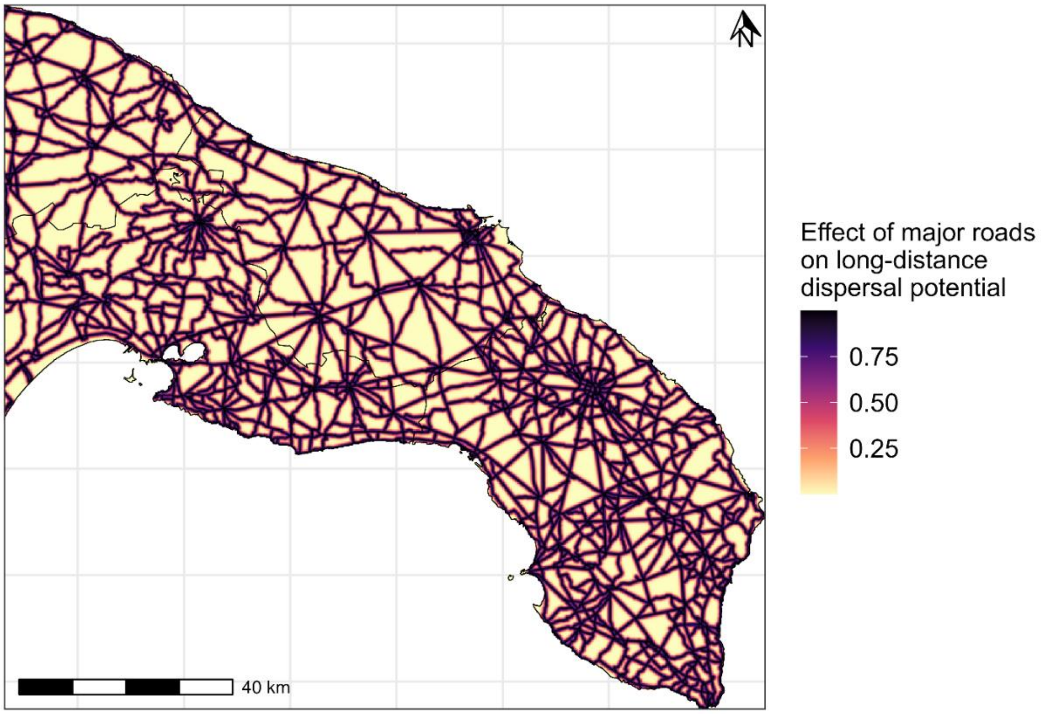

Figure D. *Illustrative effect of major roads on potential long-distance dispersal, showing the distance-decay of immigration away from roads using* m_{short} *= 0.3 km (close to its posterior median). New infection foci founded by long distance dispersal will tend to appear in olive groves within the darker shaded areas. The base map is reproduced from the GADM Global administrative areas dataset, under Creative Commons Attribution-ShareAlike 2.0 (https://gadm.org/license.html).*

S1.3. Laboratory testing model

To estimate the accuracy of diagnostic testing for *X. fastidiosa*, we extracted from the regional monitoring database records of 21,116 olive trees that recorded both their visual symptomatic status (symptomatic and asymptomatic) and had complete results of diagnostic testing. This diagnostic testing involved both Enzyme-Linked Immunosorbent Assay (ELISA) and quantitative polymerase chain reaction (qPCR), with confirmation of *X. fastidiosa* infection only through a positive qPCR result.

Test accuracies were estimated using a latent class analysis (LCA) model fitted with the poLCA R package [4] and specifying two latent classes representing infected and uninfected samples. As can be seen in Table A, LCA estimated that visual assessment was not very accurate, with very high false negative and positive rates. ELISA also had relatively high false positive rates but had a lower rate of false negatives (i.e., it had high sensitivity but lower specificity). LCA estimated qPCR to be extremely accurate and was essentially a gold standard test.

Table A. *Latent Class Analysis estimates of false negative and false positive rates and corresponding sensitivity and specificity for visual assessment of symptoms, ELISA and qPCR tests for Xylella fastidiosa infection of olive trees*

**Test**	**False negative rate**	**False positive rate**	**Sensitivity**	**Specificity**
Visual symptoms	0.4222	0.3624	0.5778	0.6376
ELISA	0.0631	0.2781	0.9369	0.7219
qPCR	<0.0001	<0.0001	>0.9999	>0.9999

All trees were tested with ELISA but not all trees were tested with the more accurate qPCR. Therefore, the overall test accuracies were largely determined by the qPCR testing rates (Table B). As can be seen, trees with a positive ELISA were nearly all tested by qPCR, while for those with negative ELISA, only around 19% of symptomatic trees and 3% of asymptomatic trees were tested by qPCR.

Table B. *Proportions of trees tested using qPCR, following visual assessment of symptoms and ELISA testing*

Visual symptoms result	ELISA result	Proportion undergoing qPCR
Positive	Positive (or dubious)	0.9979
Positive	Negative	0.1896
Negative	Positive (or dubious)	0.9942
Negative	Negative	0.0271

Based on these findings, false positives should be negligible, since false positives produced by the visual assessment and/or ELISA would be virtually all corrected by qPCR. However, false negatives can arise when infected trees receive a negative ELISA result and are not put forward for qPCR. This can be estimated as the ELISA false negative rate multiplied by the probability of a negative ELISA result not undergoing qPCR. Our estimates of the false negative rates (Z) used in the model are therefore:

- **Symptomatic trees:** Z_{symp} = 0.0631 * (1 – 0.1896) = 0.0512
- **Asymptomatic trees:** Z_{asymp} = 0.0631 * (1 – 0.0271) = 0.0614

S1.4. Bayes Factor model selection

We used Approximate Bayesian Computation (ABC) to evaluate the relative support for the three different long-distance scenarios (basic, wind and road) using Bayes Factors [5]. The three models had the same number of parameters so use of information criteria was not needed. We were specifically interested to investigate whether the two more complex dispersal scenarios (wind and road) offered a relative improvement over the basic scenario, so computed Bayes Factors to compare the wind and road models to the basic model. For this, rejection sampling was performed using 10^5 simulations for the basic and either the wind or road dispersal models. Then the proportion of accepted models from each scenario was calculated. The Bayes Factor for the wind and road models was the ratio of their acceptance proportions compared to that of the basic model, which indicates the relative support for those models versus the basic one [6]. Bayes Factor greater than $\sqrt{10}$ was interpreted as indicating substantial support for that model over the basic one [5]. To investigate the sensitivity of the Bayes Factors to the choice of acceptance threshold we calculated them over a range of thresholds.

S2. Results

S2.1. Comparison of dispersal scenarios using Bayes Factors

From 10^5 simulations, Bayes Factors for the 'road' model of long-distance dispersal compared to the basic model of isotropic long-distance dispersal were much less than one regardless of the acceptance threshold (clearly supporting the basic model over this one. Bayes Factors for the 'wind' dispersal model against the 'basic' model were >1 over all acceptance thresholds (Fig E), indicating some support for that model over basic isotropic dispersal. However, the level of support only crossed the threshold for substantial support ($BF>\sqrt{10}$) [5] for two particular acceptance thresholds (Fig E). We therefore considered that in general the support for the wind model was insufficient to select it over the conceptually simpler basic model, even though it did give a slightly better fit to the data.

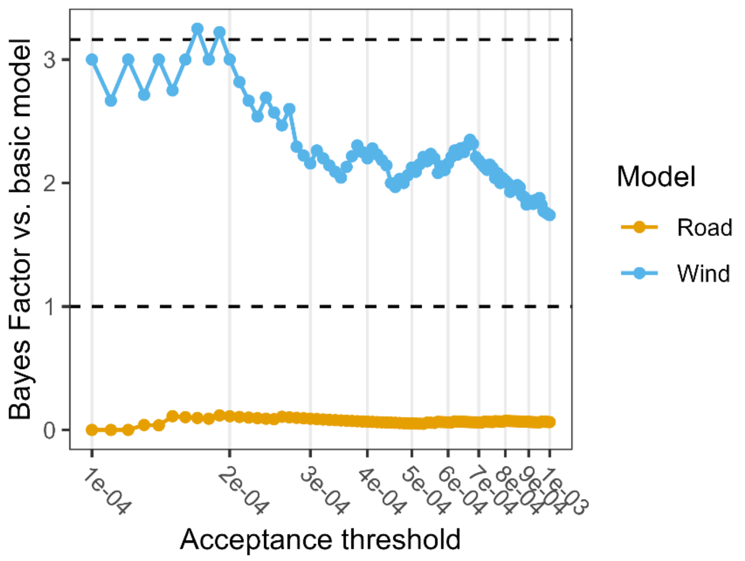

Figure E. *Bayes Factors (BF) evaluating the relative support for two long distance dispersal models (road and wind) over the basic model with isotropic dispersal, and their sensitivity to the ABC acceptance threshold used. Dashed lines show two important BF thresholds: BF < 1 indicates greater relative support for the basic model; BF > √10 indicates substantially greater relative support for the more complex model [5].*

S2.2. Posterior predictive checks

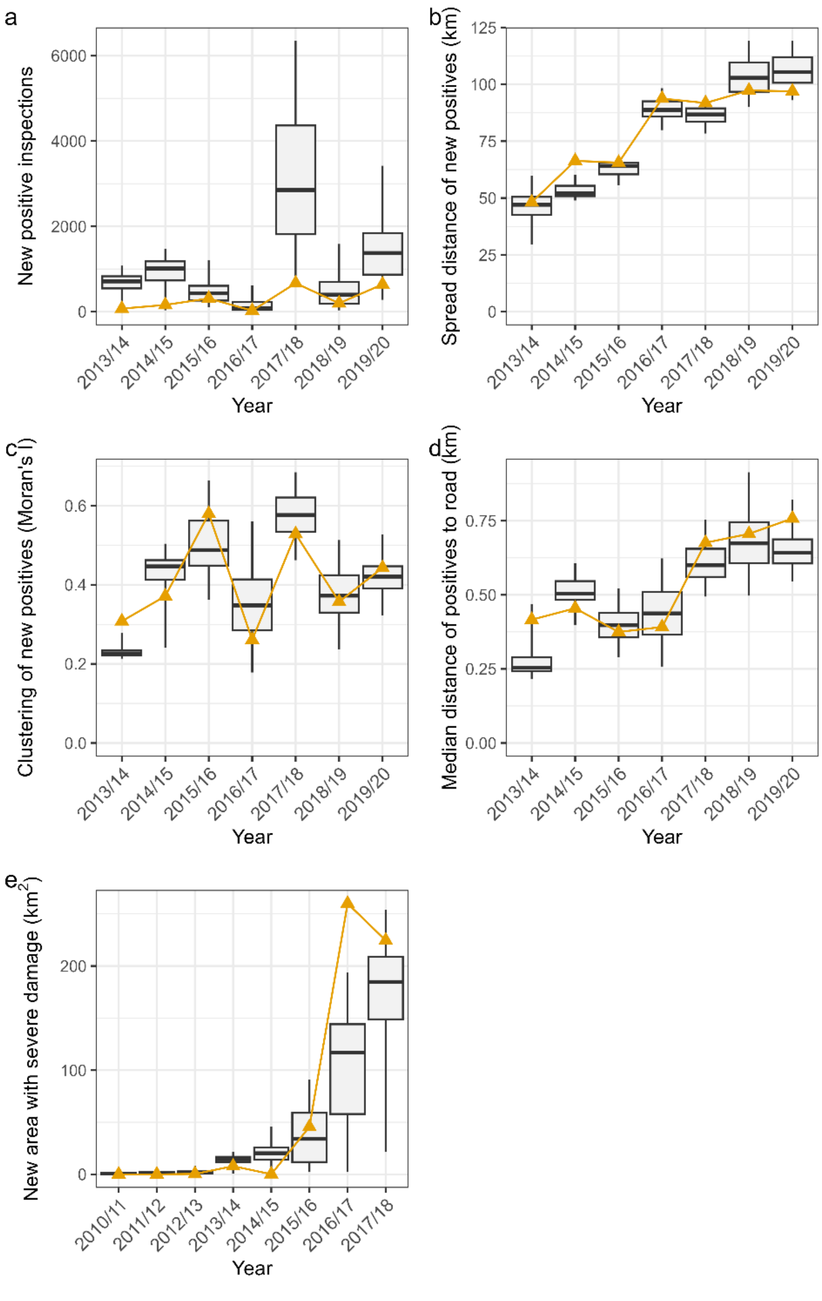

Figure F. *Posterior predictive checks contrasting the summary statistics from 100 posterior simulations (boxplots) with the observed values (triangles and lines). Boxplots show the posterior medians (thick lines), interquartile ranges (boxes) and 95% ranges (whiskers).*

S2.3. Example spread simulation

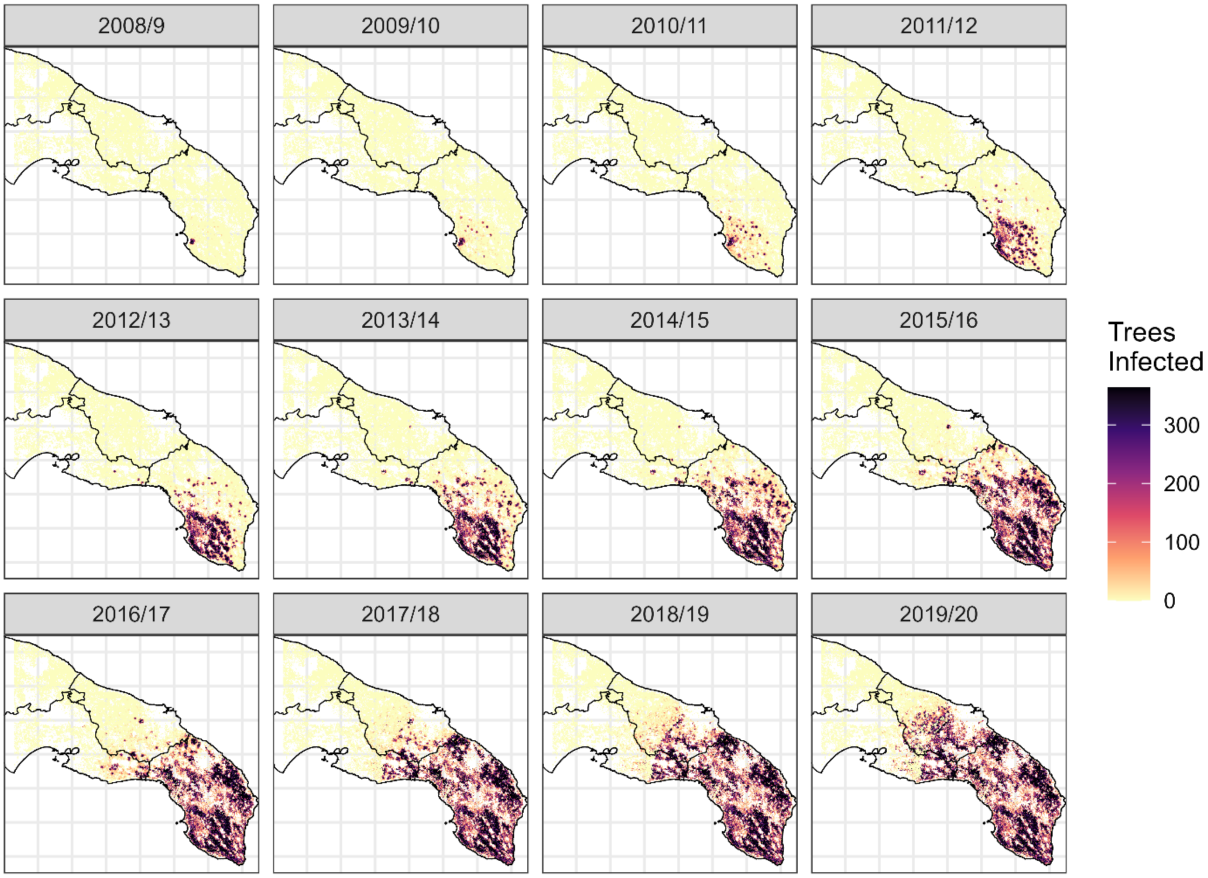

Figure G. *Example of a single simulation of the model with one set of parameters randomly drawn from the posterior distribution. The simulation shows the patchier evolution of individual modelled outbreaks (foci of new infection locations), particularly near the wave front, driven by stochastic long-distance dispersal, as compared to the smoother average behaviour shown in Fig 3 of the main text. The base map is reproduced from the GADM Global administrative areas dataset, under Creative Commons Attribution-ShareAlike 2.0 (https://gadm.org/license.html).*

Supporting References

1. Stein AF, Draxler RR, Rolph GD, Stunder BJB, Cohen MD, Ngan F. NOAA's HYSPLIT Atmospheric Transport and Dispersion Modeling System. Bulletin of the American Meteorological Society. 2015;96: 2059–2077. doi:10.1175/BAMS-D-14-00110.1

2. Iannone R. SplitR: v0.4. Zenodo; 2016. doi:10.5281/ZENODO.594806

3. Bajocco S, Raparelli E, Bregaglio S. Assessing the driving role of the anthropogenic landscape on the distribution of the *Xylella fastidiosa*-driven "olive quick decline syndrome" in Apulia (Italy). Science of the Total Environment. 2023;896: 165231.

4. Linzer DA, Lewis JB. **poLCA** : An *R* Package for Polytomous Variable Latent Class Analysis. J Stat Soft. 2011;42. doi:10.18637/jss.v042.i10

5. Kass RE, Raftery AE. Bayes factors. Journal of the American Statistical Association. 1995;90: 773–795.

6. Csilléry K, Blum MGB, Gaggiotti OE, François O. Approximate Bayesian computation (ABC) in practice. Trends in ecology & evolution. 2010;25: 410–418.
